# Balance task difficulty affects postural sway and cortical activity in healthy adolescents

**DOI:** 10.1007/s00221-020-05810-1

**Published:** 2020-04-23

**Authors:** Arnd Gebel, Tim Lehmann, Urs Granacher

**Affiliations:** 1 Division of Training and Movement Sciences, Research Focus Cognition Sciences, University of Potsdam, Am Neuen Palais 10, Building 12, 14469 Potsdam, Germany; 2 Exercise Science and Neuroscience Unit, Department of Exercise and Health, Faculty of Science, Paderborn University, Warburger Straße 100, 33098 Paderborn, Germany

**Keywords:** Balance, Postural control, EEG, Theta, Alpha-2, ICA, Youth

## Abstract

Electroencephalographic (EEG) research indicates changes in adults’ low frequency bands of frontoparietal brain areas executing different balance tasks with increasing postural demands. However, this issue is unsolved for adolescents when performing the same balance task with increasing difficulty. Therefore, we examined the effects of a progressively increasing balance task difficulty on balance performance and brain activity in adolescents. Thirteen healthy adolescents aged 16–17 year performed tests in bipedal upright stance on a balance board with six progressively increasing levels of task difficulty. Postural sway and cortical activity were recorded simultaneously using a pressure sensitive measuring system and EEG. The power spectrum was analyzed for theta (4–7 Hz) and alpha-2 (10–12 Hz) frequency bands in pre-defined frontal, central, and parietal clusters of electrocortical sources. Repeated measures analysis of variance (rmANOVA) showed a significant main effect of task difficulty for postural sway (*p* < 0.001; *d* = 6.36). Concomitantly, the power spectrum changed in frontal, bilateral central, and bilateral parietal clusters. RmANOVAs revealed significant main effects of task difficulty for theta band power in the frontal (*p* < 0.001, *d* = 1.80) and both central clusters (left: *p* < 0.001, *d* = 1.49; right: *p* < 0.001, *d* = 1.42) as well as for alpha-2 band power in both parietal clusters (left: *p* < 0.001, *d* = 1.39; right: *p* < 0.001, *d* = 1.05) and in the central right cluster (*p* = 0.005, *d* = 0.92). Increases in theta band power (frontal, central) and decreases in alpha-2 power (central, parietal) with increasing balance task difficulty may reflect increased attentional processes and/or error monitoring as well as increased sensory information processing due to increasing postural demands. In general, our findings are mostly in agreement with studies conducted in adults. Similar to adult studies, our data with adolescents indicated the involvement of frontoparietal brain areas in the regulation of postural control. In addition, we detected that activity of selected brain areas (e.g., bilateral central) changed with increasing postural demands.

## Introduction

Postural control requires the complex interaction of different structures within the somatosensory system to maintain and recover balance during the performance of sport and everyday activities (Shumway-Cook and Woollacott 2012). Several electroencephalographic (EEG) studies provide evidence that postural control involves the activity of cortical structures under static (e.g., unperturbed/perturbed upright stance) (Edwards et al. 2018; Hülsdünker et al. 2015a, b; Peterson and Ferris 2018; Slobounov et al. 2009; Solis-Escalante et al. 2019; Varghese et al. 2014) and dynamic conditions (e.g., unperturbed/perturbed walking) (Peterson and Ferris 2018; Sipp et al. 2013; Wagner et al. 2016, for a review see Wittenberg et al. 2017). Most of these studies observed altered activation that contributed to postural control across different cortical areas located near anterior cingulate, dorsolateral prefrontal cortex, supplementary motor areas, parietal, and temporal cortices on either the channel (Edwards et al. 2018; Hülsdünker et al. 2015a, b) or the source level (Peterson and Ferris 2018; Sipp et al. 2013; Solis-Escalante et al. 2019; Wagner et al. 2016). It has been shown that the application of perturbation impulses during standing and walking results in immediate power increases within the delta (1–3 Hz), theta (4–7 Hz), alpha (8–12 Hz), beta (13–24 Hz), and gamma (30–50 Hz) frequency bands (Peterson and Ferris 2018; Sipp et al. 2013; Slobounov et al. 2009; Solis-Escalante et al. 2019; Varghese et al. 2014). The broadband increment in power is presumably related to cortical processes responsible to detect postural threats. Moreover, Solis-Escalante et al. (2019) reported that broadband power increases were accompanied by concomitant multifocal increases in theta frequency band power. Accordingly, the current state of postural stability/instability could be reflected in the activity level of a cortical network that is involved in postural control (Solis-Escalante et al. 2019; Varghese et al. 2019). There is evidence that changes in the EEG power spectrum due to postural instability occur predominantly in the theta and alpha frequency bands. While previous studies established connections between theta frequency dynamics in fronto-central areas and attentional (Klimesch 1999; Sauseng et al. 2010) as well as cognitive control processes (Anders et al. 2018; Cavanagh and Frank 2014), progression in task-difficulty and postural stability/instability have also been associated with increased theta frequency band power in frontal and parietal cortical areas (Edwards et al. 2018; Hülsdünker et al. 2015a, b; Sipp et al. 2013; Varghese et al. 2014). In fact, Sipp et al. (2013) and Hülsdünker et al. (2015a) proposed that an increased fronto-central theta band power might be indicative of a postural error detection system that monitors postural stability/instability and initiates adaptive postural responses in situations of high postural instability to maintain or regain balance. In this context, Sipp et al. (2013) hypothesized that theta frequency band activity could be involved in the transfer of sensory information during the performance of postural demanding tasks. In support of this argument, studies that examined cortical activity during beam walking (Sipp et al. 2013) or the performance of different balance tasks with increasing difficulty level (Del Percio et al. 2009; Edwards et al. 2018; Hülsdünker et al. 2015b) reported a strong reactivity of the alpha frequency band in terms of decreases in power, particularly in parietal areas. While widespread fluctuations in the alpha-1 frequency band (8–10 Hz) are supposed to reflect global processes of attention and alertness (i.e., power decrease), as well as idling (i.e., power increase) (Smith et al. 1999), activity within the alpha-2 frequency band (10–12 Hz) appear to be associated with sensory and movement-related information processing (Leocani et al. 1997; Pfurtscheller et al. 1996). More specifically, there is evidence of altered alpha-2 frequency band power that is associated with task-specific cortical information processing and communication between frontal and parietal cortical structures (Bazanova and Vernon 2014).

Of note, balance task difficulty can primarily be increased by diminishing the sensory input (e.g., eyes opened/closed), by reducing the base-of-support (e.g., bipedal vs monopedal stance), by changing the characteristics of the surface (e.g., stable/unstable), or a combination of these modalities. Considering the variety of modifying factors, it is difficult to establish how these multimodal factors contribute to task difficulty. Previous research (Edwards et al. 2018; Del Percio et al. 2009; Hülsdünker et al. 2015a, b; Tse et al. 2013; Varghese et al. 2014) examined the effects of performing continuous balance tasks of varying difficulty levels on cortical activity. However, these studies either modified sensory input, base-of-support, and surface characteristics (Edwards et al. 2018; Hülsdünker et al. 2015a, b; Tse et al. 2013) or they reduced the base-of-support by changing the stance position (Del Percio et al. 2009; Varghese et al. 2014). In other studies (Dohm-Acker et al. 2008; Cimadoro et al. 2013) that changed the base-of-support only, this was done using different balance exercise tools (e.g., sissles, balance pads etc.).The use of different exercise equipment may cause bias, because the experiment is not standardized and controlled for this factor. Therefore, and in an attempt to elucidate the effects of balance task difficulty, one single factor should be addressed per study (i.e., manipulation of base-of-support OR sensory input). Moreover, if base-of-support is manipulated it should be done using one standardized balance exercise tool, while all other modalities including sensory input are kept constant. Accordingly, changes in cortical activity can solely be attributed to the systematic manipulation of base-of-support. Since scalp electrodes record a mixture of activity from distinct brain areas, the localization of these sources is mathematically undetermined (Nunez and Srinivasan 2006). Signal processing techniques such as independent component analyses (ICA) have the potential to identify maximally independent sources of functional brain dynamics. Previous studies have shown that ICA is applicable even during whole body movements such as walking or running (Gwin et al. 2010; Wagner et al. 2016). Hence, source space analyses may provide a deeper insight into the activation of cortical areas with increasing instability and postural demands. In addition, the aforementioned studies investigated only adult populations. As the brain (Arain et al. 2013) still matures during adolescence, it is uncertain whether posture-related brain activity in adolescents follows similar patterns as reported in the adult literature. Moreover, information on neurophysiological mechanisms related to postural control are hardly available for youth (Gebel et al. 2020). However, knowledge on the underlying neurophysiological correlates of postural control in youth are needed to design and develop balance training programs for the general youth population and for young athletes. Therefore, more research is needed with adolescents to elucidate frequency characteristics of cortical activity during the performance of balance tasks with increasing task difficulty.To the authors’ knowledge, there are currently no studies available that investigated how a graded increase in balance task difficulty affects cortical activity in a healthy youth population.

Therefore, the objectives of this study were to examine the effects of a gradual increase in balance task difficulty (only by changing the base of support) on postural sway and frequency band power by means of ICA-based source space analyses in healthy adolescents. Based on the relevant literature, we expected that increasing postural demands result in increased postural sway (Muehlbauer et al. 2012) and in changes of cortical activity in frontal, central, and parietal areas (Edwards et al. 2018; Hülsdünker et al. 2015a, b; Sipp et al. 2013; Slobounov et al. 2009). We further hypothesized that progression in task difficulty (i.e., reduced base-of-support) results in a concomitant increase in theta frequency band power in frontal and central areas. Of note, there is evidence that these regions of interest adopt attentional and error-related feedback processes (Hülsdünker et al. 2015a; Sipp et al. 2013; Slobounov et al. 2009; Varghese et al. 2014). Simultaneously, increasing postural demands may result in increased sensory processing reflected by decreased alpha-2 frequency band power in centro-parietal regions (Hülsdünker et al. 2015b).

## Materials and methods

### Participants

Based on the large main effect (*η*^2^ = 0.59) of base of support on theta frequency band power reported by Hülsdünker et al. (2015b), an a priori power analysis with *G* × Power (Version 3.1.9.2, University of Kiel, Germany) using a single group repeated measures analysis of variance (rmANOVA) design with 7 levels (baseline and 6 levels of task difficulty) was calculated. The analysis revealed that a total sample size of *N* = 8 would be sufficient to find significant and large-sized main effects of difficulty level (effect size *f* = 0.4, *α* = 0.05, power = 0.80), with an actual power of 0.85 (critical *F* value = 2.32). A physical education class including 13 (3 female/10 male) healthy high-school students aged 16–17 years volunteered to participate in this study. Anthropometrics as well as the results on postural sway and electromyographic activity of the leg muscles have been reported previously (Gebel et al. 2019). The EEG data presented in this article were recorded in the same study using the same study design. All participants and their legal guardians gave their written informed consent prior to the onset of the study. The study was approved by the local ethics committee of the University of Potsdam (application no. 18/2017) and conducted according to the latest version of the Declaration of Helsinki.

### Experimental procedure

A single group repeated measures design was used to examine the effects of increasing balance task difficulty on postural sway and cortical activity in healthy adolescents. For this purpose, participants attended the biomechanics laboratory for a single experimental session. Every session started with a standardized familiarization to introduce the multi-directional balance training device (balance board) and its six difficulty levels. Thereafter, participants performed three sets of six balance tasks. Each set consisted of a randomized order of the six levels of balance task difficulty. Overall, testing of one participant comprised 18 trials (3 × level 1–6) with each trial lasting 30 s per level. Continuous EEG activity was recorded during every trial, while participants performed respective balance tasks on the balance board. Furthermore, we recorded a separate 3 min EEG baseline condition during quiet bipedal stance prior to the first set and another separate baseline measure after the third set. Anthropometric data (i.e., body height and body mass) were assessed using a stadiometer (seca 213, seca Gmbh, Hamburg, Germany) and a bioimpedance analysis system (InBody 720, BioSpace, Seoul, Korea), respectively.

### Balance tasks and balance performance

**Fig. 1:**
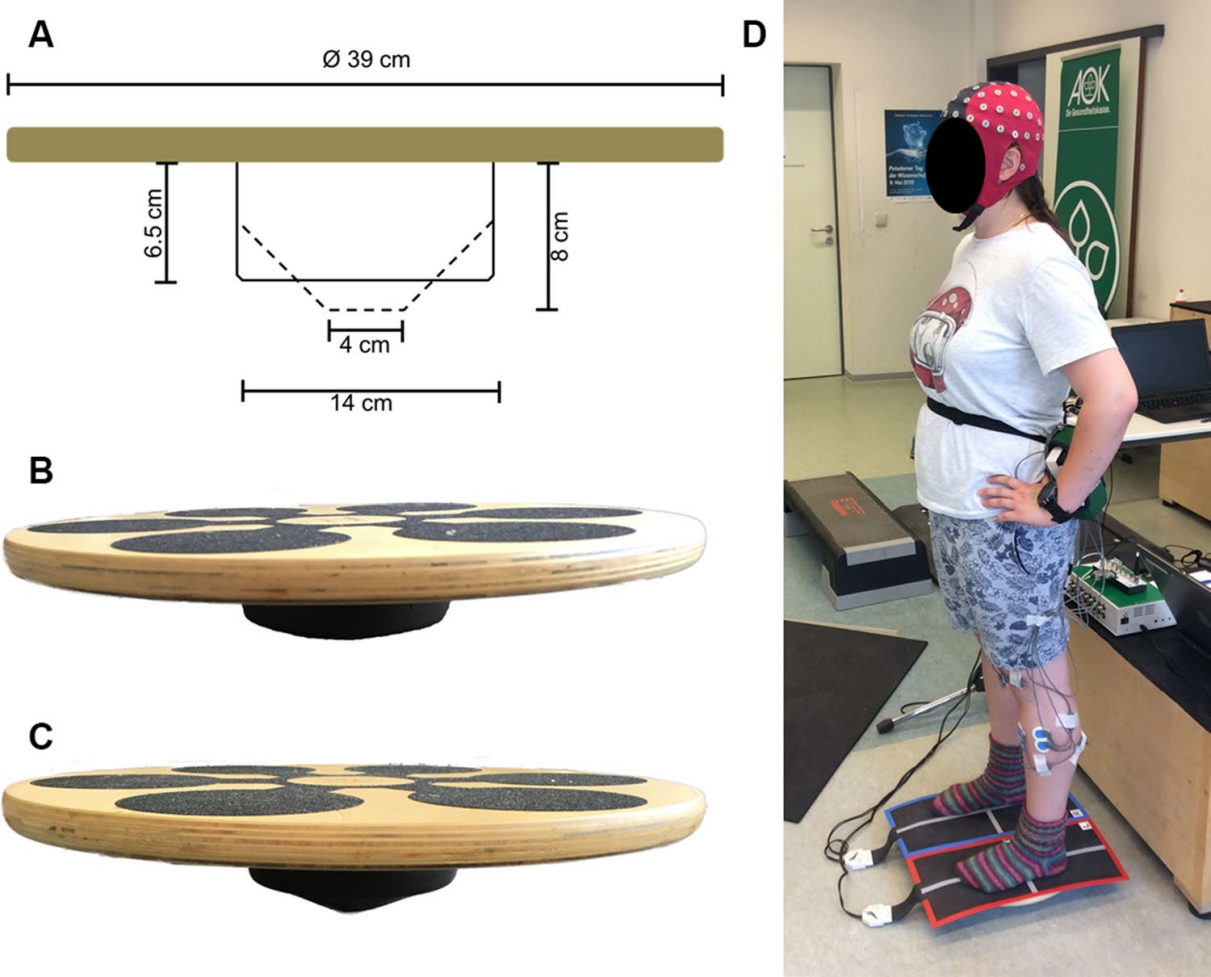
**a** Schematic representation of the balance board with its mechanically adjustable pivot modified according to Gebel et al. (2019). **b** Balance board with the pivot at level 1. **c** Balance board with the pivot at level 6. **d** Experimental setup

The applied balance tasks and the testing of balance performance were similar to our previously published study (Gebel et al. 2019). For a more detailed description of the balance tasks and the CoP data analysis, readers are referred to the methods section of Gebel et al. (2019). All balance tasks comprised bipedal upright standing (without shoes) on the balance board. Trials started from a standardized position (i.e., participants held on to a handrail in front of them) which allowed the participants a quiet stance to bring the board in horizontal position. During data recording, participants were instructed to hold their hands akimbo and to fixate their gaze at a cross on a nearby wall (3 m distance). In addition, participants were instructed to keep the balance board as steady as possible in the horizontal plane and to avoid ground contact with the board’s edges during the trials. The progressive increase in balance task difficulty was realized using a commercially available multi-directional balance board (Wobblesmart^©^, Artzt GmbH, Dornburg, Germany) which allows to tilt in every direction. The pivot attached to the board platform has an integrated mechanism to increase task difficulty. By gradual clockwise rotation, the pivot can be adjusted at six different positions (level 1–6). The change in position elevates the platform progressively from 6.5 cm (level 1) to 8 cm (level 6) and simultaneously reduces the pivots base-of-support from approximately 14–4 cm (Fig. 1a–c). During the balance tests on the balance board, postural sway (i.e., absolute CoP displacements in medio-lateral and anterior–posterior direction) was assessed as a measure for balance performance using a pressure sensitive measuring system (Pedar^©^, novel GmbH, München, Germany). For this purpose, two pressure-sensitive sensor mats (Posturo S2094, novel GmbH, München, Germany) were fixed on the balance board to record CoP trajectories at the maximum sampling rate of 40 Hz using the Posturo 32 Expert software (version 25.3.6, novel GmbH, München, Germany). EEG and CoP data were synchronized at the start of the CoP recordings by sending a continuous 5 V signal from the Pedar© system (Posturo Sync Box, novel GmbH, München, Germany) to the EEG system (Fig. 1d). Absolute CoP displacements provided by the Posturo 32 Expert software were averaged for every participant and each level of task difficulty.

### EEG recordings and analysis

Cortical activity was continuously recorded during each test condition on the balance board. EEG signals were assessed utilizing a mobile EEG system (eego™ sports, Advanced Neuro Technology B.V., Enschede, Netherlands) with 64 Ag/AgCl passive electrodes implemented in an elastic sensor cap (Waveguard classic, Advanced Neuro Technology B.V., Enschede, Netherlands). Electrode positions were set according to the extended 10–20 system of electrode placement. Channels were referenced to the CPz electrode and electrode impedance was kept below 5 kΩ to provide a high signal-to-noise ratio. The analog EEG signals were amplified and then digitized using a 24-bit analog-to-digital converter (eego™ sports, Advanced Neuro Technology B.V., Enschede, Netherlands). Digitized EEG signals were recorded with a sampling frequency of 1,024 Hz using the eego™ software (ANT Neuro eego™, Version 1.6, Neuro Technology B.V., Enschede, Netherlands).

The acquired EEG data were processed offline using MATLAB (Mathworks Inc., Natick, MA, USA) and the EEGLAB 13.5.4b toolbox (Delorme and Makeig 2004). For further analysis, at first line noise was removed with the help of the CleanLine plugin (Mullen 2012). Thereafter, physiological signals were band pass filtered with a finite impulse response filter ranging from 3 to 30 Hz and finally down-sampled to 256 Hz. Channels with electrode movement artefacts, non-stereotypical electromyographic activity and bad scalp contact were manually removed upon visual inspection. Thereafter, EEG data were re-referenced to common average. Typically, we had to discard 10 channels (± 3) per participant. Continuous data were visually inspected and the identified non-stereotypical artifacts were removed from the data set. Furthermore, data points before and after trigger onset/offset were removed. An adaptive mixture ICA (Palmer et al. 2011) was performed on the remaining data to extract spatio-temporal features of cortical activity for each participant and to identify stationary and maximally independent components (IC) (Makeig et al. 1996). Furthermore, an equivalent dipole model for each IC was calculated using a four-shell spherical head model implemented in the DIPFIT toolbox (Oostenveld and Oostendorp 2002). According to the heuristic approach as described by Onton and Makeig (2006), we separated functional activity from stereotypical artifacts. This means that ICs were rated as functional by visual inspection on the basis of the scalp topographic maps, time courses, frequency spectra, and location of the dipole model. ICs that were rated as functional were considered for further analyses. However, ICs with artifacts from electro-oculographic (i.e., eye blinks) sources and muscle electromyographic activities were dismissed. If the single equivalent dipole model of a functional IC revealed more the 15% residual variance from the spherical four-shell head model, the component was also rejected from further analyses. A *k* means algorithm was applied to cluster the remaining ICs across all participants. ICs were assigned to a cluster if they were located within two standard deviations of the respective cluster. Clusters that contained components from less than 11 participants (< 80% of the sample) were excluded Overall, 170 ICs were used for cluster analysis with an average of 13 ICs (± 4) per participant. For frequency specific analyses, EEG data were merged for all three trials within a level of task difficulty. This was done for each participant separately. After artefact rejection and IC identification, the average length of the merged trials was 86.2 s (± 6.2 s) per level. Absolute spectral power was calculated for two predefined frequency bands (4–7 Hz [theta], 10–12 Hz [alpha-2]) and for each IC using a fast Fourier transformation with a spectral resolution of 1 Hz and a 10% Hanning window. For analyses, absolute spectral power for each frequency band was averaged across individual ICs within the respective clusters.

### Statistical analyses

All statistical tests were performed using SPSS (Version 25, IBM, Chicago, IL, USA). Model residuals of CoP and EEG data were tested using the Shapiro–Wilk test to verify normality assumption for repeated measures analyses of variance (rmANOVA). To control if balance performance was affected by task difficulty, a single rmANOVA was computed for postural sway (absolute CoP displacements) with the six levels of task difficulty as repeating factors. Furthermore, seven separate rmANOVAs were computed for the absolute spectral power of predefined frequency bands (theta and alpha-2) within the respective clusters. The factor task difficulty comprised six increasing difficulty levels together with baseline measures. If significant main effects of task difficulty were registered for balance performance or cortical activity, post-hoc tests were applied using Bonferroni-corrected paired *t* tests. Thus, it was possible to identify differences between single levels of balance task difficulty in both balance performance as well as cortical activity for each cluster. If necessary, the Greenhouse–Geisser correction for non-sphericity was applied. The level of significance was set at *p* ≤ 0.05 for all statistical analyses. Effect estimates of partial eta-squared (*η*_p_^2^) were converted into Cohen’s *d* and interpreted according to Cohen (1988) with ≥ 0.2 as small, ≥ 0.5 as medium, and ≥ 0.8 as large effects.

## Results

### Balance performance

The rmANOVA results for postural sway indicated a significant main effect of task difficulty (*F*_(2.4, 29.4)_ = 121.6, *p* < 0.001; *d* = 6.36). Post-hoc tests showed a significant increase in CoP displacements with increasing task difficulty. A more detailed report on the post-hoc results including figures can be found in a previous study (Gebel et al. 2019).

### Cortical sources

**Fig. 2:**
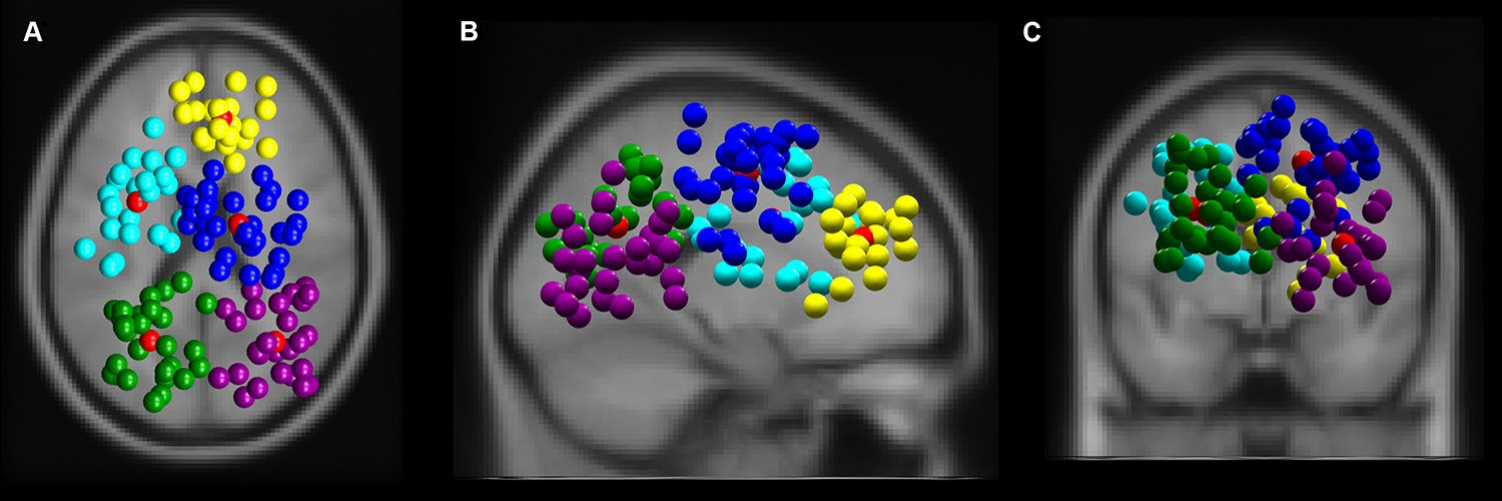
Clusters of independent component EEG sources localized in frontal (yellow), central left (cyan), central right (blue), parietal left (green), and parietal right (purple) from top (**a**), sagittal (**b**), and coronal view (**c**). Red spheres indicate respective cluster centroids. All other colored spheres indicate a single EEG signal source

The *k* means clustering algorithm revealed five robust clusters composed of electrocortical sources in frontal (*n*_IC_ = 21, 11 participants), bilateral central (central left *n*_IC_ = 22, 13 participants, central right (*n*_IC_ = 32, 11 participants) and bilateral parietal (parietal left *n*_IC_ = 29, 11 participants, and parietal right (*n*_IC_ = 26, 13 participants) areas (Fig. 2a–c).

### Theta frequency band

**Fig. 3:**
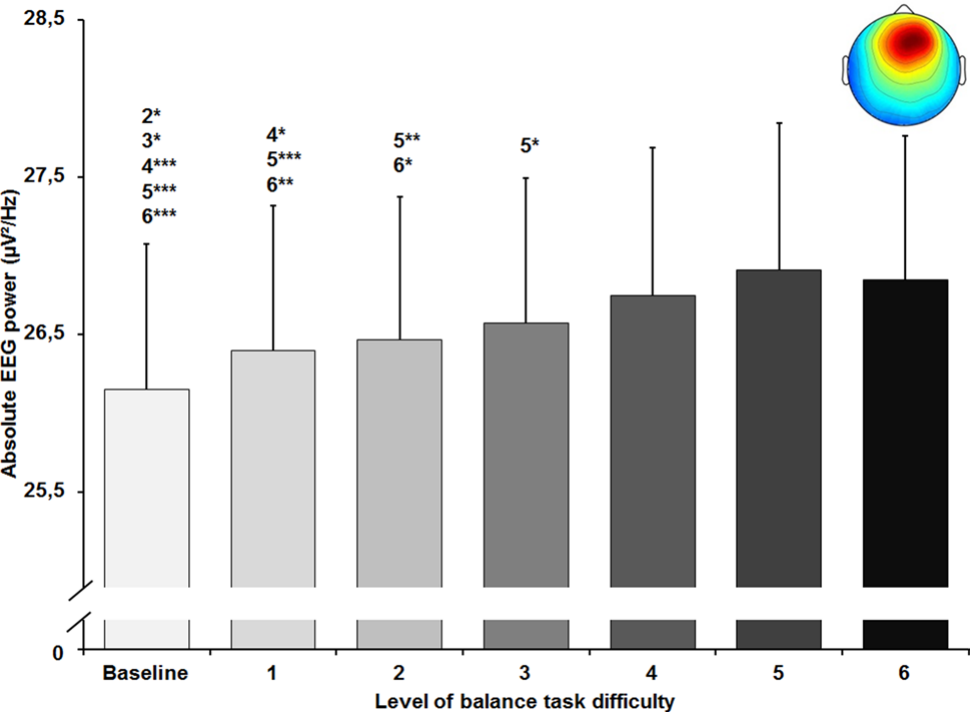
Absolute theta frequency band power in µV^2^/Hz in the frontal cluster (scalp map in the upper right corner) with standard error of the mean across all six levels of balance task difficulty. Significant differences between levels are indicated by level number with respective asterisks; *p < 0.05, **p < 0.01, and ***p < 0.001

The rmANOVA revealed a significant large-sized main effect of balance task difficulty for absolute frontal theta frequency band power (*F*_(6, 120)_ = 16.137, *p* < 0.001; *d* = 1.80). Post hoc tests identified a significant increase in absolute theta power (Fig. 3). The increment in power was significant between baseline and levels 2–6 (all *p* values ≤ 0.017), between level 1 and levels 4–6 (all *p* values ≤ 0.05), between level 2 and levels 5 and 6 (all *p* values ≤ 0.023) and between level 3 and level 5 (*p* = 0.029). Effect sizes of the applied post-hoc tests ranged between *d* = 0.07–0.17.

**Fig. 4:**
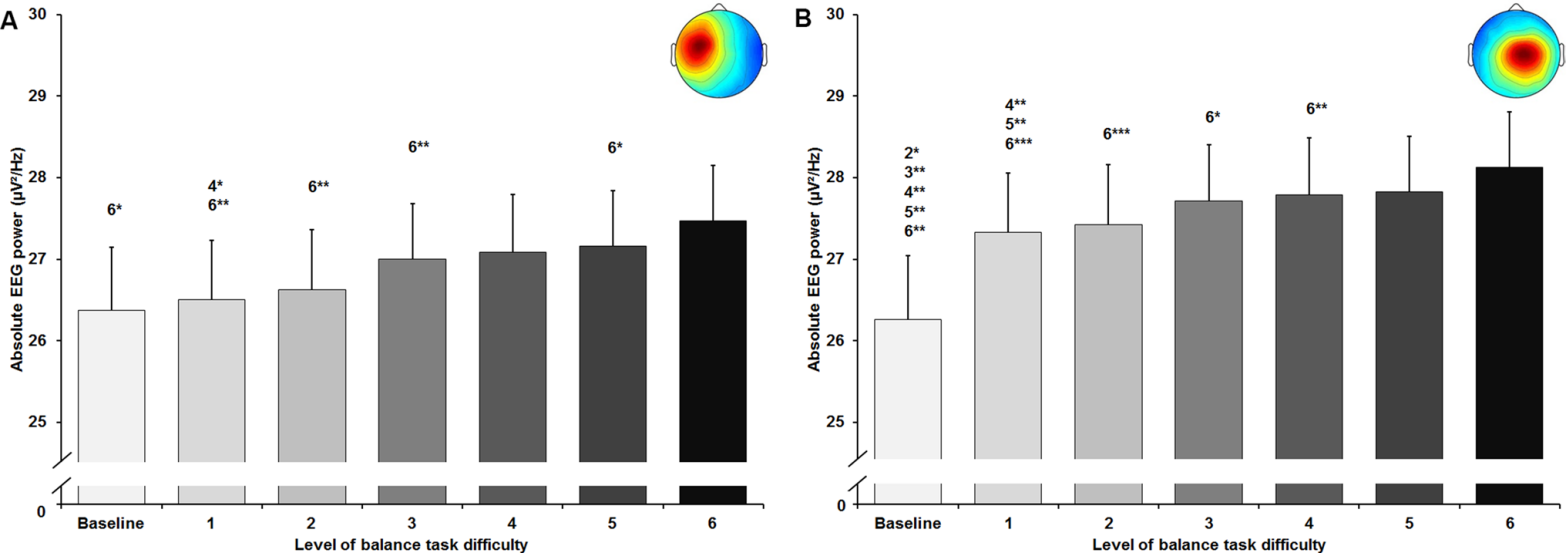
Absolute theta frequency band power in µV^2^/Hz in the central left (**a**) and central right (**b**) cluster (respective scalp maps in the upper right corner) with standard error of the mean across all six levels of balance task difficulty. Significant differences between levels are indicated by level number with respective asterisks; *p < 0.05, **p < 0.01, and ***p < 0.001

A significant main effect of task difficulty was found for absolute theta frequency band power in the central left (*F*_(1.84, 38.56)_ = 11.594, *p* < 0.001; *d* = 1.49) and in the central right (*F*_(1.50, 46.64)_ = 15.637, *p* < 0.001; *d* = 1.42) cluster as well. Post-hoc tests revealed significant increases in absolute theta power for the central left cluster (Fig. 4a) between level 6 and all other levels of task difficulty (all *p* values ≤ 0.043, 0.09 ≤ *d* ≤ 0.31), except level 4 which differed significantly from level 1 (*p* = 0.016; *d* = 0.17). Significant increments in power were found for the central right cluster (Fig. 4b) between baseline and levels 2 to 6 (all *p* values ≤ 0.029, 0.37 ≤ *d* ≤ 0.57), between level 1 and levels 4–6 (all *p*-values ≤ 0.009, 0.13 ≤ *d* ≤ 0.22), as well as between levels 2 to 4 and level 6 (all *p* values ≤ 0.012, 0.09 ≤ *d* ≤ 0.2).

### Alpha-2 frequency band

**Fig. 5:**
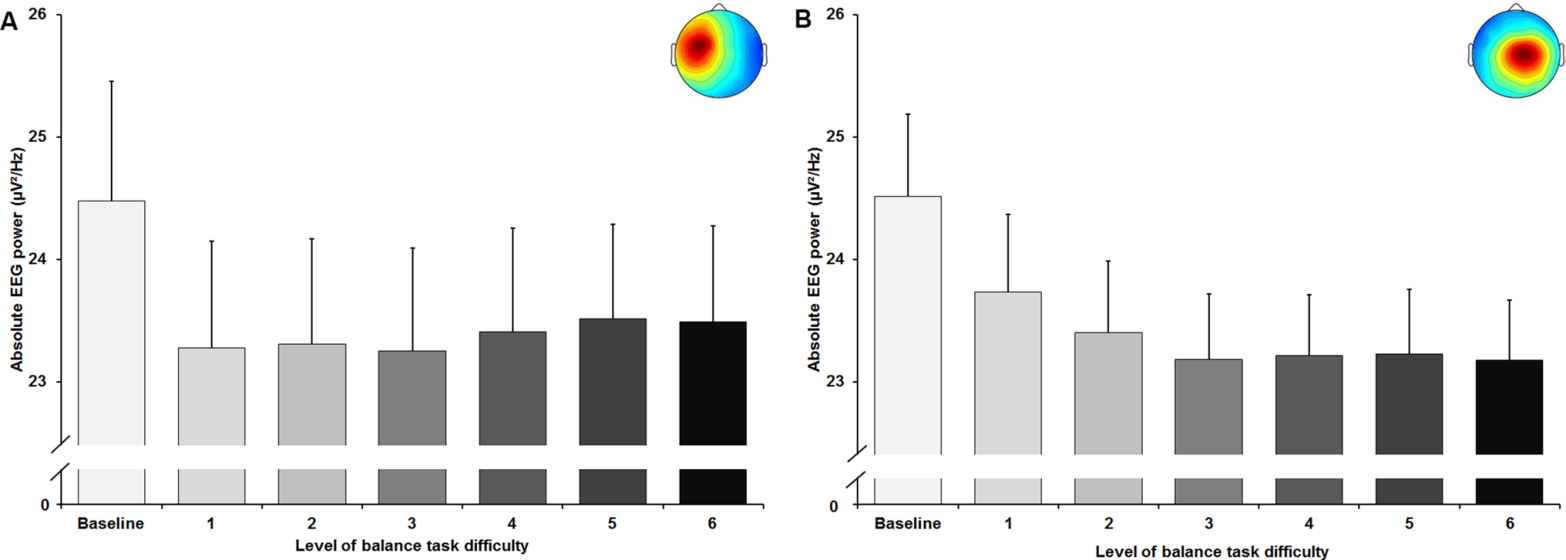
Absolute alpha-2 frequency band power in µV^2^/Hz in the central left (**a**) and central right (**b**) cluster (respective scalp maps in the upper right corner) with standard error of the mean across all six levels of balance task difficulty. Significant differences between levels are indicated by level number with respective asterisks; *p < 0.05, **p < 0.01, and ***p < 0.001

**Fig. 6:**
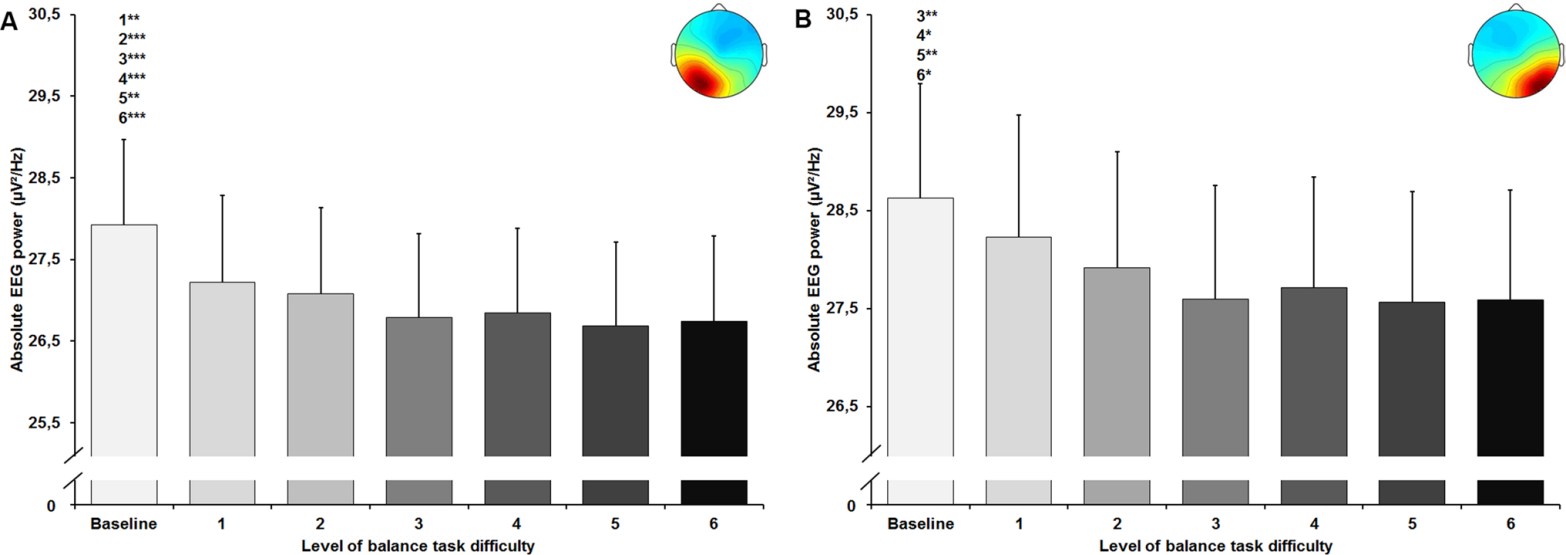
Absolute alpha-2 frequency band power in µV^2^/Hz in the parietal left (**a**) and parietal right (**b**) cluster (respective scalp maps in the upper right corner) with standard error of the mean across all six levels of balance task difficulty. Significant differences between levels are indicated by level number with respective asterisks; *p < 0.05, **p < 0.01, and ***p < 0.001

The statistical analyses for central brain areas showed a significant large-sized main effect of balance task difficulty for absolute alpha-2 frequency band power in the centralR cluster (*F*_(1.62, 50.15)_ = 6.632, *p* = 0.005; *d* = 0.92). All applied post-hoc tests did not reach the level of significance (Fig. 5a). No significant main effect was found for the centralL cluster (*F*_(1.64, 34.39)_ = 2.755, *p* = 0.087; *d* = 0.72) (Fig. 5b). Furthermore, both clusters in the parietal area showed a significant large-sized main effect of task difficulty for absolute alpha-2 power (parietalL *F*_(2.60, 72.78)_ = 13.614, *p* < 0.001; *d* = 1.39; parietalR *F*_(2.94, 73.38)_ = 6.885, *p* < 0.001; *d* = 1.05). For the parietal cluster (Fig. 6a), post-hoc tests identified significant decreases in power between baseline and levels 1 to 6 (all *p*-values ≤ 0.003, 0.12 ≤ *d* ≤ 0.22). For the parietalR cluster (Fig. 6b), significant decreases were found between baseline and levels 3 to 6 (all *p* values ≤ 0.028, 0.15 ≤ *d* ≤ 0.18).

## Discussion

This study is the first to examine cortical activity in the theta and alpha-2 frequency bands using ICA analyses while performing a balance task with a progressively increased task difficulty level in healthy adolescents. The main findings of this study were that postural sway (i.e., CoP displacements) increased and cortical activity changed with increasing balance task difficulty. In terms of cortical activity, theta frequency band power in frontal and bilateral central (left and right) areas increased with increasing balance task difficulty. Furthermore, we found significant decreases in alpha-2 frequency band power with increasing instability in bilateral parietal (left and right) areas. Alpha-2 power in bilateral central areas decreased as well but we observed no significant differences between levels of task difficulty.

### Balance performance

In accordance with our hypothesis, we observed increased postural sway when task difficulty increased. Our findings in adolescents are consistent with previous studies (Donath et al. 2016; Muehlbauer et al. 2012) investigating how an increasing balance task difficulty affects balance performance in adults. Donath et al. (2016) as well as Muehlbauer et al. (2012) reported an increase of postural sway with increasing task difficulty in young (Donath et al. 2016; Muehlbauer et al. 2012) and old adults (Donath et al. 2016). In contrast to these studies, which manipulated stance (e.g., bipedal, unipedal) and sensory inputs (i.e., surface, vision), we only reduced the balance boards’ base of support to increase task difficulty. Considering the implementation of a progressive increase of task difficulty into a balance training protocol, our results suggest that an increase in task difficulty by reducing the base of support of a balance board has a more continuous slope than manipulating several external conditions (i.e., stance, vision) simultaneously. Furthermore, the reduction of the base of support also has an impact on neuromuscular activity. Using the same study design, Gebel et al. (2019) recently reported that these decreases in balance performance were accompanied by increases in lower limb muscle activity and muscle coactivation. The authors interpreted their findings as a change in the underlying postural strategy caused by increasing postural demands. This statement should be verified in future studies using cortico-muscular coherence analysis.

### Theta frequency band

As hypothesized, we found significant increases of theta frequency band power in frontal and central areas with increasing balance task difficulty in adolescents. These findings are consistent with previous studies in healthy adults which observed an increase in theta power over frontal (Hülsdünker et al. 2015a, b) and central electrode sites (Edwards et al. 2018; Hülsdünker et al. 2015a, b) during continuous balance tasks with varying degrees of instability. These authors suggested that the increase in theta power over frontal and central electrodes may originate from the anterior cingulate cortex and sensorimotor areas, which are highly involved in processes of error detection and sensory information processing (Slobounov et al. 2009; Varghese et al. 2014). A few other studies reported increases in frontal and central theta power induced by sudden postural perturbations during quiet bipedal stance (Varghese et al. 2014), unipedal stance (Slobounov et al. 2009), and while walking on a balance beam (Sipp et al. 2013). Furthermore, Sipp et al. (2013) showed that walking on a narrow balance beam compared to treadmill walking resulted in increased theta power in cortical sources located near or in anterior cingulate cortex, anterior parietal, dorsolateral prefrontal, and sensorimotor cortex. In this context, attentional processes responsible for successful balance performance under challenging conditions may contribute to increased theta power in frontal areas. Loss of balance appears to be associated with immediate increases in theta power across multiple cortical areas including the anterior cingulate and anterior parietal cortex (Sipp et al. 2013). After balance recovery, activity even decreased below baseline level. These authors hypothesized that the observed increase in theta band activity may act as an error detecting system to initiate situation-specific postural responses. The existence of a balance-specific cortical network has further been supported by findings from Solis-Escalante et al. (2019) who observed increased theta power in the anterior cingulate, prefrontal, posterior parietal, sensorimotor cortex, and supplementary motor area following the application of perturbation impulses during bipedal standing. These authors interpreted the multifocal theta power enhancement as activity of a cortical network that is involved in detecting postural threats and initiating adequate postural responses. Moreover, Varghese et al. (2019) speculated upon the existence of a cortical balance control network. Their assumption was based on widespread topological rearrangements in functional cortical connectivity within delta, theta, alpha, and beta frequency bands during the performance of reactive balance tasks. Our results may point in a similar direction and can be interpreted as activation of a balance control network during the performance of a continuous balance task. Therefore, the continuous increase in theta frequency band power within the frontal and central clusters may reflect a higher information processing load due to increased level-dependent postural demands.

As previous studies in adults, our analyses of the frontal cluster showed no further increase of theta frequency band power in the balance task with highest level of difficulty. This may be referred to as a “ceiling effect” (Edwards et al. 2018; Hülsdünker et al. 2015a), demonstrating no further increase in theta band spectral power when instability becomes excessive and postural demands are too high to maintain balance. However, in contrast to Hülsdünker et al. (2015a) and Edwards et al. (2018), we observed this phenomenon only in the frontal cluster and not in both central clusters, where theta power further increased in the highest level of balance task difficulty. This “ceiling effect”, restricted to frontal areas, might be also explained by other processes than postural error detection. Findings of numerous studies associated higher frontal theta power with increased attention in cognitive (Smith et al. 1999), visuomotor (Slobounov et al. 2000), complex motor (Baumeister et al. 2008), and sensorimotor tasks (Baumeister et al. 2013). Based on their findings, Smith et al. (1999) as well as Baumeister et al. (2008) suggested that changes in frontal theta power are related to focused attention as well as engagement and effort into a specific task. In this context, observed increases in frontal theta power may indicate increased focused attention and the concomitant activation of additional attentional resources when the difficulty of the balance task increased. However, if the demands of the balance task exceed the individuals’ capability to maintain balance, focused attention and the allocated attentional resources remain at the same level due to consistent task-specific engagement and effort. This suggestion was supported by the fact that at the highest level of difficulty most participants were unable to move the balance board back into the horizontal plane after leaving the start position. Participants tilted the board from edge to edge. Otherwise, this position could have offered more stability for the participants and, therefore, afforded less attentional resources than balancing on the narrow pivot of the balance board. However, this would arise the question why theta power further increased in sensorimotor areas despite increased stability and decreased sensory information being processed.

### Alpha-2 frequency band

In line with our hypothesis, we found reductions in alpha-2 frequency band power in both central and parietal areas at both hemispheres when balance task difficulty increased. Whereas increases in alpha power are considered to reflect inhibitory processes in task-irrelevant brain areas to facilitate information processing in task-relevant areas, decreases in alpha-2 power indicate task-specific information processing (Del Percio et al. 2007, 2009; Klimesch et al. 2006; Slobounov et al. 2009). Previous studies reported decreased power in the alpha frequency band with increasing balance task difficulty in electrode-based regions of interest (Edwards et al. 2018; Hülsdünker et al. 2015b). For instance, Hülsdünker et al. (2015b) reported that decreases in alpha-2 power were strongest in centro-parietal areas (CP1, CPz, CP2, P3, Pz, P4) with increasing instability. The authors interpreted their findings as increased sensory information processing caused by compensatory postural movements. Furthermore, Edwards et al. (2018) observed similar alterations in the broad alpha frequency band (8–12 Hz). They reported decreased alpha power over centro-parietal electrode sites (C3, Cz, C4, P3, P4) with increased level of task difficulty and suggested that information processing increased with balance challenge. In addition, results of Sipp et al. (2013) showed decreased frequency band power between 8 and 12 Hz (alpha-1 and alpha-2) in functional clusters located near sensorimotor cortex of both hemispheres when walking on a balance beam compared to treadmill walking in young adults. In this context and in view of the present results, significant reductions in alpha-2 power, predominantly in bilateral parietal areas, may suggest an incremental functional involvement of both areas in postural control processes with increasing task difficulty and instability. This assumption may be further supported when considering the level-dependent development of the alpha-2 power in both parietal clusters found in the present study. Even though significant differences in alpha-2 frequency band power were only observed between baseline and higher levels of balance task difficulty, alpha-2 power seemed to further decrease from the lowest (levels 1–2) to the highest levels (levels 5–6) (Fig. 6a, b). Interestingly, we observed such tendencies between the lowest and highest levels of difficulty not for both central but the central right cluster. As reductions of alpha-2 power in sensorimotor areas were associated with increased processing of sensory and movement-related information (Leocani et al. 1997; Pfurtscheller et al. 1996), present results of decreases in mainly parietal alpha-2 power may indicate increased sensory and movement-related information processing with increasing instability.

### Limitations

A methodical limitation of this study is the approach of source space localization by means of only 64 EEG channels. We are aware of the inversion problem when conducting source space analyses. Therefore, results of the ICA-based source space localization should be interpreted with caution, since precise localization of cortical activity is only possible with high-density EEG systems, co-registration, and additional functional magnetic resonance imaging. However, as minimal standard source space analyses require at least an EEG system with 64 channels for data acquisition, although precision increases with the number of channels used (Sohrabpour et al. 2015). Furthermore, an ICA does not necessarily separate all relevant components for each participant. It is also possible that multiple ICs from one participant represent a single source and contribute to the same cluster. This may affect the statistical analyses. Here, we used all ICs, because we could not rule out that multiple ICs represent a single source but with different time-dependent characteristics. Furthermore, this approach has frequently been used in the literature (Peterson and Ferris 2018; Sipp et al. 2013; Solis-Escalante et al. 2019; Wagner et al. 2016). Currently, there is no consensus on how to deal with this problem. Another limiting or confounding factor might be the continuous visual input throughout the experiment. In contrast to studies of Hülsdünker et al. (2015b) and Edwards et al. (2018) who observed a reduction in alpha-2 power during balance tasks without visual input, the participants in our study performed all balance tasks with eyes opened. Since we kept visual conditions (i.e., gaze fixation at a cross at 3 m distance) constant during every level of balance task difficulty, it is more likely that decreases in alpha-2 power were a result of somatosensory information processing.

## Perspectives

In summary, the present study revealed decreased balance performance (i.e., postural sway) as well as frequency characteristics of cortical activity on basis of ICA-based source space analyses evoked by a continuous increase in balance task difficulty in healthy adolescents. Consistent with previous adult studies, we found increased theta frequency band power in frontal and central clusters reflecting attentional and error-based processes as well as decreased alpha-2 frequency band power, mainly in parietal areas, reflecting sensory information processing as a function of increasing postural demands and task difficulty. These findings support the notion that frontal, bilateral central as well as parietal areas are involved in postural control processes with increasing postural demands which may reflect the activity of cortical balance network. Furthermore, we demonstrated that EEG source localization can be applied during a continuous balance task with increasing level of difficulty. Moreover, we were able to show that postural control strategies involve the activity of frontal, bilateral central as well as parietal brain areas and that activity of these areas change with increasing postural demands. Therefore, future studies may use high-density EEG systems to specify these functional areas and their time–frequency characteristics during increasing instability as well as cortico-muscular coherence analysis to link cortical to muscle activation patterns during increasing postural demands.
